# Pre-Diagnostic Circulating Vitamin D and Risk of Melanoma in Men

**DOI:** 10.1371/journal.pone.0035112

**Published:** 2012-04-25

**Authors:** Jacqueline M. Major, Christine Kiruthu, Stephanie J. Weinstein, Ronald L. Horst, Kirk Snyder, Jarmo Virtamo, Demetrius Albanes

**Affiliations:** 1 Division of Cancer Epidemiology and Genetics, National Cancer Institute, National Institutes of Health, Bethesda, Maryland, United States of America; 2 Heartland Assays, Inc., Ames, Iowa, United States of America; 3 Information Management Services, Inc., Silver Spring, Maryland, United States of America; 4 Department of Chronic Disease Prevention, National Institute for Health and Welfare, Helsinki, Finland; Brigham & Women's Hospital, and Harvard Medical School, United States of America

## Abstract

**Purpose:**

Various studies have examined the association between serum vitamin D levels and different cancers; however, this is the first prospective study of this association with melanoma risk. The aim of this study is to investigate the association between serum vitamin D [25(OH)D] levels and melanoma in a cohort of older, middle-aged Finnish male smokers.

**Methods:**

We conducted a nested case-control study within the Alpha-Tocopherol Beta-Carotene Cancer Prevention (ATBC) Study. From the ATBC cohort, 368 subjects were chosen for our study; 92 participants that developed melanoma and 276 matched control subjects. At study baseline, lifestyle questionnaires and blood samples were collected. Serum 25(OH)D was modeled as three sets of categorical variables: clinically-defined categories, season-specific quartiles and season-adjusted residual quartiles. Conditional logistic regression was used to obtain odds ratios (ORs) and 95% confidence intervals (95% CIs) to estimate the association between circulating vitamin D and melanoma risk.

**Results:**

Overall no association of serum 25(OH)D and melanoma risk was observed. A decreased risk of developing melanoma was observed in the middle categories compared to the lowest category, albeit not significant.

**Conclusion:**

Results indicate no association between serum 25(OH)D levels and melanoma. Additional studies, including possibly consortium efforts, are needed to investigate the association between serum 25(OH)D levels and risk of melanoma in larger, more diverse study populations.

## Introduction

Skin cancer is the most common cancer in the United States, with malignant melanoma being the most lethal and clinically invasive form resulting from transformation of melanocytes in skin (ocular and intestinal primaries also occur) [1]. The incidence of melanoma has more than doubled in the past 20 years, and among men, it is the fifth most common cancer in the U.S. [2]. Risk of developing melanoma increases with age and is higher among men and fair-skinned individuals; one in 39 Caucasian men develops melanoma in their lifetime. The primary etiologic factor for melanoma is solar ultraviolet (UV) radiation exposure [3]–[5], but interest in the potential role of circulating high vitamin D concentrations has increased owing in part to recently observed associations with reduced melanoma tumor progression and improved survival [6]–[8].

Vitamin D status is contributed to by ultraviolet B (UVB) exposure from sunlight and by diet and supplements [9], [10]. Vitamin D is first metabolized in the liver to 25-hydroxyvitamin D [25(OH)D], and then in the kidney and other organs to its active form, 1,25-dihydroxycholecalciferol [1,25(OH)D]. 1,25(OH)D is essential to calcium homeostasis and functions by regulating cell proliferation and differentiation in various organs [11]. Circulating levels of 25(OH)D serves as a marker of vitamin D status based on its accessibility and precursor function to hormonal form, 1,25(OH)D [12], [13]. Although some prospective studies suggest that high levels of circulating 25(OH)D may be associated with decreased risk of colorectal cancer, and possibly breast cancer [9], the relation with incident melanoma is unclear. In human melanoma cell lines, in vitro studies indicate 1,25(OH)D inhibits cell growth [14], [15]. Case-control studies have found associations with improved prognosis, but no association between 25(OH)D and disease status based on post-diagnostic blood samples [6], [16]. There is a need, therefore, to further examine this relation in prospective studies in which serum vitamin D levels were measured prior to disease development. The purpose of this study was to examine the association between prediagnostic serum vitamin D [25(OH)D] and subsequent risk of melanoma in a prospective study of older, middle-aged Finnish men.

## Materials and Methods

### Study population

We conducted a nested case-control study within the Alpha-Tocopherol, Beta Carotene Cancer Prevention (ATBC) Study cohort. The present analysis included 368 men; 92 participants that developed melanoma and 276 matched (3∶1 ratio) control subjects to provide sufficient power to detect an odds ratio of 0.50 at 80% power (alpha = 0.05). Incident cases, as defined by the International Classification of Diseases 9 code (172, melanoma), were diagnosed from April 1986 through April 2005. All melanoma cases were identified through the Finnish Cancer Registry, which provides close to 100% population-based cancer ascertainment in Finland. Controls were selected from ATBC Study participants that were alive at the time of the case diagnoses, and were matched to each case based on age (+/−3 years) and date of baseline serum collection (+/−30 days).

The ATBC Study was a randomized, controlled trial that examined whether supplementation with alpha-tocopherol (vitamin E) or beta-carotene would reduce the cancer incidence in male smokers. Details have been previously reported [17], but in brief, the study was conducted in southwestern Finland and included 29,133 male smokers between the ages of 50–69 years, who smoked at least five cigarettes a day, had no recent history of cancer, severe illnesses (e.g., cirrhosis of liver, chronic renal insufficiency, or other medical problems), or use of predefined doses of vitamin E, A or beta carotene. The study was reviewed and approved by the institutional review boards of the National Public Health Institute in Finland and the U.S. National Cancer Institute. All study participants provided written informed consent.

### Study measures

At study entry, investigators administered questionnaires that collected information on medical history and lifestyle behaviors, including diet and smoking history. Height and weight were measured using standard methods, and body mass index (BMI, kg/m^2^) was calculated. A follow-up questionnaire was administered 3 years after baseline which ascertained information on skin behavior (e.g., more prone to sunburn) and sun exposure.

#### Laboratory assays

Serum was collected at study entry, stored at −70°C, and 25(OH)D levels were analyzed using the LIAISON 25-OH Vitamin D Total Assay (DiaSorin, Inc., Stillwater, MN) at Heartland Assays, Inc. (Iowa). Matched case and control samples were masked to case status and assayed in batches. Quality control samples were 5% of the total number of samples. Intrabatch and interbatch coefficients of variation (CV) ranged between 9.3%–11.0% and 12.3%–13.6%, respectively.

### Statistical analysis

Descriptive statistics were calculated for baseline characteristics of participants by cancer status. The distribution of baseline characteristics between cases and controls were compared using the Wilcoxon rank-sum test for continuous variables and Chi-square test for categorical variables.

Serum 25 (OH)D was modeled as three distinct categorical variables: clinically-defined categories (<25, 25 to 37.49, 37.50 to 49.99 and 50+ nmol/L), season-specific quartiles, and quartiles of season-adjusted residuals. The season-specific quartiles of 25 (OH)D were based on the distribution among control subjects within the “darker” (November–April) and “lighter” (May–October) months. The quartiles for the residuals of the season-adjusted 25(OH)D values were obtained using a weighted polynomial regression and modeling on log-transformed 25(OH)D levels that adjusted for date of blood draw [18]. Conditional logistic regression was used to obtain odds ratios (ORs) and corresponding 95% confidence intervals (CIs) to estimate the association between vitamin D levels and melanoma risk. Potential confounders and variables associated with 25(OH)D levels and/or melanoma were included in the initial model building process. Variables that changed the ORs by 10% or more were considered as potential confounders. Final parsimonious models adjusted for age (continuous), date of serum collection, skin behavior (burns easily: no/yes/missing), dietary cholesterol (continuous), height (continuous), and weight (continuous). Effect modification of cigarette smoking, BMI, height, leisure physical activity, or trial supplementation was examined through inclusion of a cross-product term in the model. Data were analyzed using SAS® (version 9.2, SAS Institute Inc., Cary, NC) and all *P*-values were two-sided.

## Results

Melanoma cases were taller, heavier, and more likely to have at least an elementary school education than the controls (Table 1). By contrast, cases had lower dietary intake of cholesterol and fish and were less likely to have reported their skin type and whether or not they had taken trips to the south. Serum 25(OH)D levels were similar for cases and controls. The median age at diagnosis was 65 years; mean (standard deviation) was 66 (7.1) years. The median time from baseline blood draw to diagnosis was 8.9 years. The median follow-up time for the control subjects was 18.2 years.

**Table 1:** Baseline characteristics of men, ATBC Study.

Characteristic	Cases (N = 92)		Control (N = 276)		*P* -value*
Age at randomization (yrs)	57.5	(53.5–61.0)	57.0	(53.5–61.0)	0.97
Education>elementary school, n (%)	30	(32.6)	59	(21.4)	0.03
Married, n (%)	16	(17.4)	40	(14.5)	0.51
Height (cm)	176.0	(171.0–179.0)	173.0	(169.0–177.0)	<0.01
Weight (kg)	84.8	(77.8–90.5)	77.8	(70.1–86.1)	<0.01
BMI (kg/m^2^)	26.5	(24.7–29.2)	26.1	(23.6–28.4)	0.07
Dietary Intake (per day)					
Cholesterol (mg)	507.5	(380.4–596.9)	546.3	(417.5–738.3)	0.03
Energy (kcal)	2549.1	(2228.3–2953.9)	2691.7	(2224.8–3145.9)	0.21
Fish (g)	29.3	(19.9–45.3)	35.4	(22.1–54.5)	0.07
Total vitamin D (µg)	4.2	(3.1–6.4)	5.1	(3.6–6.5)	0.07
Total calcium (mg)	1426.0	(1056.1–1724.2)	1419.1	(1002.3–1794.7)	0.79
Alcohol intake (g)	88.1	(26.3–250.0)	91.7	(17.1–253.8)	0.98
Smoking history					
No. cigarettes/d	20.0	(15.0–25.0)	20.0	(15.0–23.5)	0.42
No. years smoked	38.0	(30.5–42.0)	35.0	(30.0–42.0)	0.33
Physical activity-leisure, n (%)					0.95
Light	36	(39.1)	107	(38.8)	
Moderate/heavy	56	(60.9)	169	(61.2)	
Skin burns easily†, n (%)					<0.01
No	40	(43.5)	170	(61.6)	
Yes	30	(32.6)	86	(31.2)	
Missing	22	(23.9)	20	(7.2)	
Trips south-daily sun exposure ≥1 hr, n (%)					<0.01
None	42	(45.7)	141	(51.1)	
≥1 trip	28	(30.4)	118	(42.8)	
Missing	22	(23.9)	17	(6.2)	
Serum draw months‡, n (%)					0.89
Light months	30	(32.6)	87	(31.5)	
Dark months	62	(67.4)	189	(68.5)	
Serum 25(OH)D (nmol/L)	33.1	(21.9–51.6)	31.8	(20.9–48.5)	0.56

Medians (25^th^, 75^th^ percentiles) are reported unless otherwise indicated.

* *P*-value for contrast between cases and controls by Wilcoxon rank sum test (medians) and Chi-square test (proportions).

† Skin behavior in prolonged direct sunlight (self-reported).

‡ Light months = May–October, dark months = November–April.

Information on skin behavior and sun exposure was ascertained from a follow-up questionnaire which was missing for some men.

We observed no association between serum 25(OH)D levels and risk of melanoma (Table 2; *Ptrends*>0.05). For clinically-defined categories, lower risk of melanoma was suggested for serum vitamin D levels between 37.50 to 49.99 nmol/L, but then an increased risk of melanoma for men whose vitamin D levels were ≥50 nmol/L (ORs = 0.60 and 1.32, with corresponding 95% CIs = 0.25–1.44 and 0.64–2.72, respectively) when compared to men whose prediagnostic levels were <25 nmol/L. Similarly, season-specific quartiles showed a decreased risk of melanoma in the second quartile (OR = 0.79; 95% CI = 0.34–1.81) and an increased risk in the third quartile (OR = 1.33; 95% CI = 0.62–2.85) when compared to the lowest quartile, albeit not statistically significant. Season-adjusted residuals, however, suggested an increased melanoma risk in the second and third quartiles (ORs = 1.20 and 1.56 with 95% CIs = 0.55–2.64 and 0.74–3.28, respectively). Tests for non-linear trends were not statistically significant (*P-*values>0.05, data not shown), and there were no significant interactions between circulating vitamin D levels and the number of cigarettes smoked per day, BMI, height, leisure physical activity, or trial supplementation (data not shown).

**Table 2:** Adjusted odds ratios of melanoma by categories of serum 25(OH)D levels.

	Categories of 25(OH)D levels (nmol/L)				
	Cat 1	Cat 2	Cat 3	Cat 4	*P* *
Clinically-defined categories					0.51
Range of values (nmol/L)	<25.00	25.00–37.49	37.50–49.99	≥50.00	
Cases/controls	32/96	23/67	11/53	26/60	
OR (95% CI)	1.00 (ref)	1.04 (0.52–2.12)	0.60 (0.25–1.44)	1.32 (0.64–2.72)	
Season-specific quartiles					0.49
Range of values (nmol/L)					
Darker months	<20.30	20.30–29.34	29.35–44.85	≥44.86	
Lighter months	<24.84	24.84–35.78	35.79–52.89	≥52.90	
Cases/controls	23/69	15/69	29/69	25/69	
OR (95% CI)	1.00 (ref)	0.79 (0.34–1.81)	1.33 (0.62–2.85)	0.98 (0.44–2.17)	
Season-adjusted residuals quartiles				0.57	
Range of values	<3.12	3.12–3.49	3.50–3.89	≥3.90	
Cases/controls	20/69	20/69	31/69	21/69	
OR (95% CI)	1.00 (ref)	1.20 (0.55–2.64)	1.56 (0.74–3.28)	0.96 (0.43–2.17)	

OR, odds ratio; CI, confidence interval.

Conditional logistic regression models adjust for age at randomization, date of blood draw, height, weight, dietary cholesterol, and skin behavior (e.g., skin burns easily in prolonged direct sunlight (no/yes/missing)).

Darker months = November–April; lighter months = May–October.

* *P*-values based on test for trend.

## Discussion

The present study is the first to prospectively examine prediagnostic serum vitamin D concentrations in relation to subsequent risk of melanoma. Our findings indicate no statistically significant association between serum 25(OH)D levels and melanoma, although there was a suggested protective association in the second quartiles compared to the lowest levels.

A previous population study examining vitamin D and non-melanoma skin cancer (NMSC) showed high serum 25(OH)D levels related to decreased risk of NMSC [19], while a case study of 14 melanoma patients found 25(OH)D serum levels ranged from 50 to 125 nmol/L, not supporting the hypothesis that deficient levels are associated with the development of malignant melanoma [20]. Our results are similar to those observed in a case-control study showing median levels of serum 25(OH)D in melanoma patients not significantly different from those of the controls (42 vs. 47 nmol/L; *P* = 0.44) [16]. That study did, however, find median levels to be significantly lower in patients with advanced melanoma (stage IV, metastasized) than those with stage Ia/b (33 vs. 41 nmol/L; *P*<0.01). Because we did not have disease stage available, we were unable to examine potential associations between serum vitamin D levels and risk of advanced melanoma in the present analysis. Although previous prospective studies have not examined risk of developing melanoma, associations between serum vitamin D level and cancer risk have been reported, with a potential U-shaped association suggested for prostate and pancreatic cancer [11], [21]. Reports from population-based studies examining higher dietary or supplemental intake of vitamin D have been inconsistent; some showing a reduced risk of melanoma overall [22], [23] and some showing no association [24], [25]. It should be noted, however, that dietary intakes of vitamin D are notoriously difficult to assess and may not reflect accurate vitamin D status.

A strength of our study is having examined serum 25(OH)D and melanoma in a prospective cohort ensuring that our vitamin D measure preceded the development of melanoma by up to 18 years. In addition, cases were identified through the Finnish Cancer Registry with approximately 100% case ascertainment. Controls were matched to the cases by age and month of blood collection, which likely reduced misclassification of vitamin D status due to seasonal variation. A limitation of the study is a lack of generalizability of the findings given that our study population consists of older middle-aged Finnish male smokers; median age at diagnosis was 65 years. We only had a single measure of serum 25(OH)D, however, which may not reflect long-term exposure and did not allow us to examine changes in vitamin D concentration levels over time although moderate correlations between measures collected years apart have been reported elsewhere [26], [27]. The interpretation of the results is limited by the small number of cases (n = 92), which may have made detection of a significant associations difficult. Further, we acknowledge that the Finnish population used in the present analysis are known to have low levels of serum 25(OH)D compared to U.S. study populations for several reasons, including the substantially higher latitude of Finland (60 degrees N), low dietary and minimal supplemental intakes of vitamin D among the ATBC Study participants, and the fact that blood samples were not collected during peak summer vacation times (mid- June through August) because the study clinics were effectively closed. It should be mentioned, however, that there is ongoing debate regarding the definition of “sufficient” levels, with consensus not yet reached. For example, in the recent IOM report, the committee's review of data suggests that persons are at risk of deficiency at serum 25(OH)D levels of below 30 nmol/L. Our cases and controls had median serum values of approximately 32 nmol/L.

In conclusion, prediagnostic circulating vitamin D concentrations were not associated with subsequent risk of melanoma. Additional prospective studies are needed to investigate the association between serum 25(OH) levels and risk of melanoma in study populations that include nonsmokers and women, and ideally, with a larger number of melanoma cases and data for stage at diagnosis.
